# Use of pre-hospital medication in children presenting with malaria to the emergency unit of Mulago Hospital, Uganda: A descriptive study

**Published:** 2017-07-01

**Authors:** Victor S. Tumukunde, Elizabeth Kiboneka, Chandy C. John, Robert O. Opoka, Richard Idro

**Affiliations:** 1 Department of Paediatrics and Child health, Mulago National Referral Hospital and Makerere University, Kampala, Uganda.; 2 Division of Global Paediatrics, University of Minnesota, USA

## Abstract

**Background:**

Initiation of specific antimalarial treatment within 24 hrs of fever onset at home and before presentation to the hospital is one of the strategies to reduce mortality from malaria in sub-Saharan Africa. In order to determine whether this strategy is being implemented we describe the use and factors associated with the use of pre-hospital medications among children admitted with malaria in one of the tertiary hospitals in Uganda.

**Materials and methods:**

Use of pre-hospital medications was assessed in 205 children aged 6-59 months and diagnosed with malaria at admission in Mulago hospital. Data were obtained on the type, source, and dose adequacy of medicines used before presentation to the hospital as well as the socio-demographical characteristics of the children. The proportion of children using pre-hospital medication was determined and logistic regression analysis used to determine factors associated with use of pre-hospital medication.

**Results:**

Overall, 147/205 (72%) of the children were given some medication for their illness before presentation to the hospital. The common pre-hospital medicines used were paracetamol (107/147, 72.8 %) and antimalarial medicines (91/147, 61.9 %). Antibiotics were used in only 12 (8.2 %) of the cases. The majority (62/91, 68%) of the cases got medicines from a health facility but only 41/91 (45%) received an adequate dose. Having fever for more than three days was significantly associated with use of pre-hospital medicines (OR = 2.2; 95% CI 1.12-4.35; p = 0.02).

**Conclusions:**

The pre-hospital use of medicines is common amongst children presenting with malaria to this tertiary Ugandan hospital. The practice is, however, associated with use of inadequate doses of antimalarials and delay in presentation to the hospital. More effort is therefore needed to educate communities on the importance of proper home management of malaria.

## 1 Introduction

In malaria-endemic regions of Africa, the majority of caretakers of children with febrile illnesses recognise fever as an early sign of malaria [1-3], often resulting into initiation of treatment at home [4-5]. Thus, in sub-Saharan Africa, 50-75% of febrile illnesses are managed at home [6-9]. If appropriate, this prompt treatment of fever should result in reduction in morbidity and mortality due to malaria or other febrile illnesses [5]. However, treatment provided at home is often inappropriate [5,10] with only as low as 15% of febrile illnesses being treated appropriately [9,11-12]. Herbal remedies and over the counter medicines, such as antipyretics, also feature prominently in treatments given at home [13-15]. The source of antimalarial medicines given at home sometimes includes leftovers from a previous episode of fever, medicines given by neighbours or from unlicensed drug shops [11]. Thus, home treatment of febrile illnesses can potentially lead to administration of inappropriate dosages and/or wrong medicines.

The prevailing practices of home use of medicines for febrile illnesses suspected to be malaria among children presenting to a tertiary setting is poorly described in Uganda. We reviewed the pre-hospital use of medicines amongst children presenting with febrile illness/suspected malaria at Mulago hospital to determine usage, types, sources and factors associated with use of pre-hospital medications.

## 2 Materials and methods

### 2.1 Study design and setting

This was a descriptive cross-sectional study conducted in the Acute Care Unit (ACU), the pediatric emergency unit of Mulago National Referral & Teaching Hospital in Kampala, Uganda. Mulago is a 1500-bed hospital that serves the urban and peri-urban population of Kampala and surrounding districts. The ACU receives all sick children 0-12 yrs of age presenting to the hospital with a medical condition requiring admission or specialist care. Each day, the unit handles 100-200 children, up to 60% of who are hospitalised. All children presenting to the ACU with fever or history of fever get a blood smear done for malaria parasites in the unit laboratory. Sick children requiring admission are stabilised and kept overnight in the unit before transfer to other general wards for further care the following morning.

### 2.2 Study participants and procedures

Using consecutive sampling, we included children 6-59 months with laboratory-confirmed malaria that presented at the ACU with febrile illness between October 2013 and January 2014. Children with sickle cell anaemia were excluded from the study.

Children presenting to ACU with febrile illness had a medical history and physical examination done, including a history of medications taken prior to presentation. A blood slide for malaria parasites was obtained from each study participant, however quantitative determination of the parasite density was not performed. Data were obtained on the type, source, and dose adequacy of the medicines used prior to presentation, the social demographic and clinical features at admission. A dosing chart based on body weight, with the recommended doses for different antimalarial medicines, was used to measure dose adequacy. In addition, samples of the different common medicines were used to ascertain the type of medicines taken before presentation to hospital. All patients were managed as per Mulago hospital guidelines that are based on WHO guidelines. The School of Medicine Research and Ethics Committee of Makerere University and the National Council of Science and Technology granted ethical approval for the study. Written informed consent for participation was obtained from all caretakers of the children studied.

### 2.3 Data analysis

Data was entered in Epi Data 3.1 database and analysed using SPSS version 19. The proportions using the different medications and the adequacy of the dose was determined. Bivariate analyses were done to explore the relationship between several independent variables and outcomes. Categorical data were compared using the Chi-square test, while the means were used for normally distributed continuous data. Variables with p-values ≤ 0.2 were then entered into multivariate logistic regression analysis to determine factors independently associated with pre-medication use.

## 3 Results

The majority of study participants were from Kampala or Wakiso districts and most of the primary caretakers, 160/201 (79.6%), were mothers. The median distance from home to the nearest public health facility was 2 km with the interquartile range of 1-4 km (Table 1). Fever was a presenting complaint in all 205 patients and 100 had severe malaria (Table 2). Of the 100 patients with severe malaria, 65 were severely anaemic, 21 had malaria with respiratory distress, 17 had malaria with repeated convulsions, and 14 had cerebral malaria. Other common presentations among patients with uncomplicated malaria were cough at 47/105 (45%), vomiting at 26/105 (25%), and diarrhoea at 17/105 (16%).

**Table 1:** Demographic and clinical characteristics of the study participants (n=205).

Characteristic	Frequency (%)	Mean, median
No. of children	205	-
Male children, no. (%)	116 (56.5)	-
Mean age, months (SD)	-	28.6 (13.9)
Mother's education level		
Primary & below	109 (53.7)	-
Secondary & above	94 (46.3)	-
Median distance from a health unit, km	-	2.0
Distance to health unit Interquartile range, km	-	1-4
Mean haemoglobin, g/dl (SD)	-	7.2 (3.2)
Level of anaemia by WHO category (%)		
Severe (Hb* <7g/dl)	100 (49.2)	-
Moderate (Hb≥7-9.9g/dl)	54 (26.6)	-
Normal (Hb >11g/dl)	30 (14.8)	-
Mild (Hb =10-10.9g/dl)	19 (9.4)	-
Nutrition status, weight/height z-score (%)		
Normal	87 (44.8)	-
-2SD*	40 (20.6)	-
-1SD	36 (18.6)	-
-3SD	31 (16.0)	-

*SD (standard deviation), Hb (Haemoglobin), g/dl (grams per decilitre). WHO reference for Hb and Nutrition status used.

**Table 2:** Symptoms and signs at presentation in the hospital.

Variables	Frequency (%)
History (n=205)	
Fever	204 (99.5)
Cough	90 (43.9)
Convulsions	53 (25.9)
Tea-coloured urine	11 (5.4)
Examination (n=205)	
Pulse rate ≥ 120 bpm	153 (74.6)
Temperature ≥37.5^o^C	115 (56.1)
Respiratory distress	21 (10.2)
Coma	12 (5.8)
Bed side lab (n=199)	
Haemoglobin ≤5g/dl	66 (33.2)
Diagnosis (n=205)	
Uncomplicated malaria	105 (51.2)
Severe malaria	100 (48.8)

The majority of patients enrolled, 147/205 (72%), had used some form of medication prior to presentation. The common types of medicines used were paracetamol 107/147(72.8%), anti-malarial medicines 91/147(61.9%) and unspecified herbal medicines 36/147(24.5%; Table 3). Out of the 91 patients who took antimalarial medicines, 49 (54%) used artemether-lumefantrine (AL) with 22/49 (45%) taking adequate doses. Quinine was used by 42 (46%) of which 18/42 (43%) used adequate doses. Overall less than half (45%) of those who used antimalarial medicines took adequate doses.

**Table 3:** Pre-hospital treatment of fever.

Variable	Frequency (%)
No. children who received treatment (n=205)	147 (71.7)
Type of medicines used (n =147)	
Paracetamol	107 (72.8)
Anti-malarials	91 (61.9)
Used more than one medicine	85 (57.8)
Herbal medicines	36 (24.5)
Antibiotics	12 (8.2)
Sources of antimalarial medicines used (n=91)	
Health facility	62 (68)
Other sources	20 (22)
Over the counter	9 (10)

The majority of antimalarial medications, 62/91(68%), were obtained from lower-level health units and clinics. Drugs bought over the counter from drug shops and pharmacies constituted 9/91 (10%). Other sources of medication were from neighbours and leftovers from previous illness (Table 3).

The baseline characteristics (age, sex, and distance from health facility) were similar among the children who received pre-hospital medicines and those that did not (Table 4). Logistic regression analysis was done to examine the factors that might be associated with use of pre-hospital medicines. At bivariate analysis, duration of fever was associated with pre-hospital use of medicines. At multivariate analysis, duration of fever of more than three days was found to be independently associated with pre-hospital medicines with an adjusted odds ratio of 2.5 (95% CI 1.3-4.7, p=0.007) (Table 4).

**Table 4:** Factors associated with use (n=147) or absence (n=58) of pre-hospital treatment.

Variable		Pre-hospital treatment		Unadjusted			Multivariate-adjusted		
		Yes n (%)	No n (%)	OR	95%CI	p-value	AOR	95% CI	p-value
Age (months) Sex	≤24	68 (46.3)	29 (50.0)	0.86	0.47-1.58	0.63	-	-	-
	>24-59	79 (53.7)	29 (50.0)						
	Male	89 (60.5)	27 (46.6)	1.762	0.95-3.25	0.07	1.17	0.63-2.20	0.616
	Female	58 (39.5)	31 (53.4)						
Fever	≤3days	68 (46.3)	40 (69.0)						
	>3days	79 (53.7)	18 (31.0)	2.58	1.36 – 4.92	0.004	2.46	1.28-4.73	0.007
Distance to facility Cough	≤5km	128 (87.1)	53 (91.4)	1.57	0.56 – 4.43	0.391	-	-	-
	>5km	19 (12.9)	5 (8.6)						
	Yes	65 (44.2)	27 (46.6)						
	No	82 (55.8)	31 (53.4)	1.099	0.60-2.02	0.762	-	-	-
House wife	Yes	60 (44.1)	28 (50.0)	0.789	0.42-1.47	0.458	-	-	-
	No	76 (55.9)	28 (50.0)						
Mother’s education	≤ primary	84 (57.9)	25 (43.1)	1.818	0.98-3.36	0.057	0.61	0.32-1.14	0.123
	≥ secondary	61 (42.1)	33 (56.9)						

When only receipt of antimalarial medicines was considered as pre-hospital treatment, duration of fever more than three days and malaria severity were each associated with pre-hospital treatment at bivariate analysis. Duration of fever more than three days and malaria severity remained associated with pre-hospital antimalarial treatment at multivariate analysis (Table 5).

**Table 5:** Factors associated with use of anti-malaria medicines prior to presentation in the hospital.

Variable	Category	Anti-malaria medicine		Unadjusted			Adjusted		
		Yes (n=91)	No (n=114)	OR	CI	p-value	AOR	CI	p-value
Age (months)	>24	50 (55.0)	58 (50.9)	1.18	0.68-2.05	0.562			
Sex	Male	57 (62.6)	59 (51.8)	1.56	0.89-2.74	0.119	1.53	0.83-2.82	0.17
Severe malaria	Yes	58 (63.7)	42 (36.8)	3.01	1.70-5.30	<0.001	2.35	1.28-4.30	0.006
Fever duration	>3 days	57 (62.6)	40 (35.1)	3.10	1.75-5.50	<0.001	2.74	1.49-5.03	0.001
Cough	Yes	39 (42.9)	53 (46.5)	0.86	0.50-1.50	0.603			
Distance to facility	>5 km	13 (14.3)	11 (9.7)	1.56	0.66-3.67	0.305	-	-	-
House wife	Yes	39 (46.4)	49 (45.4)	1.04	0.59-1.89	0.884	-	-	-
Mother's education	≤primary	55 (61.1)	54 (47.8)	1.72	0.98-3.01	0.059	1.42	0.78-2.60	0.25

## 4 Discussion

We found that nearly three quarters of children presenting to Mulago hospital with malaria had taken some form of pre-hospital treatment. Significantly among those who took antimalarials, less than half received an adequate dose.

The level of use of pre-hospital treatment of fever found in our study is similar to what has been reported by a similar study done in the region [12]. The studies that found higher levels (87.7%-96%) than reported were community or outpatient based studies [9,16]. Taken together, these findings confirm the high use of medicines in the community, which is not surprising given that these medicines are readily available as over the counter medication. The intended purpose of this is that such easy access would allow patients to get medicines early in the course of disease and prevent progression of disease to states that require referral to tertiary health facilities.

Disturbingly, however, only 61.9% of the patients had used antimalarials of which less than half (45%) had taken adequate doses. Other studies have found even lower rates of 13.2%-15.2% [9,12,16] of children receiving adequate treatment of fever at home. In addition to the inadequate doses, caregivers reported use of chloroquine and sulphadoxine-pyrimethamine, which are no longer efficacious in our setting [17,18]. Twenty four percent of the study participants had used herbal medicines for treating fever. Use of herbs is associated with delays to seek appropriate treatment which can potentially lead to disease progression [5,7,9,11,19]. Such rampant use of inappropriate doses, ineffective antimalarials and inappropriate medicines is likely to lead to treatment failure of uncomplicated malaria and development of resistance to antimalarial medicines and progression to severe forms of the disease [20]. There is therefore an urgent need to intensify health education in communities on the use of pre-hospital medicines to avoid undermining malaria control efforts.

The only factor that was independently associated with use of pre-hospital medicine was fever of more than 3 days at presentation to the hospital. Children who received pre-hospital treatment were two times more likely to present after three days of fever than those who did not receive pre -hospital treatment. Nshakira *et al.* [12] found in a community study that patients who received pre-hospital medications took longer to present to health facilities than those who did not (3.9 versus 2.6 days) [12]. Unlike other studies [21-23] the delay in this study was not because of distance from the nearest public health centre, which was found not to be associated with use of pre-hospital medicines.

The use of a specific population with confirmed malaria to study pre-hospital treatment of fever was the strength of this study. The limitations of the study included recall bias of the type of medicines used and the exact medicine doses and dose schedules adhered to, validity of history given by the caregivers [24]. We addressed this by showing medicine samples of the commonly used medicines in order to ascertain the type of medicines used. The other limitation is that we did not specifically ask for the intention of taking the medicines before presentation to hospital. However, previous studies reported that 90% of urban caretakers of children perceived malaria as the cause of fever [25].

## 5 Conclusions

The use of pre-hospital medicines is common among children with malaria presenting to tertiary hospitals like Mulago. The practice is however associated with use of inadequate doses of antimalarial medicines and presentation to the hospital after three days of fever. More effort is needed to educate communities on the importance of proper home management of malaria.
